# Supplementary material for: Optimized assay for transposase-accessible chromatin by sequencing (ATAC-seq) library preparation from adult Drosophila melanogaster neurons
Source: Sci Rep. 2022 Apr 11;12:6043. doi: 10.1038/s41598-022-09869-4 (PMC9001676; doi:10.1038/s41598-022-09869-4)
Supplement: Supplementary file 1 — Supplementary Table 1. [file 41598_2022_9869_MOESM1_ESM.docx]

**Supplementary Table 1.** Quality metrics of sequenced libraries

| Sample | # of reads | Sequencing depth (after de-duplication) | % Duplication | # of identified peaks |
| --- | --- | --- | --- | --- |
| TH1 | 87,707,383 | 30.5 | 51.1 | 69464 |
| TH2 | 87,213,024 | 30.3 | 69.8 | 69940 |
| GABA1 | 68,274,382 | 23.8 | 48.2 | 84051 |
| GABA2 | 65,981,532 | 22.9 | 43.1 | 81634 |
| GABA3 | 87,472,228 | 30.4 | 45.4 | 107215 |
