# Supplementary material for: Optimized assay for transposase-accessible chromatin by sequencing (ATAC-seq) library preparation from adult Drosophila melanogaster neurons
Source: Sci Rep. 2022 Apr 11;12:6043. doi: 10.1038/s41598-022-09869-4 (PMC9001676; doi:10.1038/s41598-022-09869-4)
Supplement: Supplementary file 2 — Supplementary Information. [file 41598_2022_9869_MOESM2_ESM.docx]

**Supplementary File 1. Optimized protocol for ATAC-seq library preparation from adult *Drosophila* neurons**

FLY LINES

UAS-Stinger-nls flies (BL84277, Bloomington Drosophila Stock Center)

TOI-Gal4 driver line (target of interest, expressing in specified neuronal population)

REAGENTS

BSA, 100 g (A30075-100.0, Research Products International)

PBS (10010023, ThermoFisher)

Tween-20 (89411-094, VWR)

NP40 (492016, Sigma Aldrich)

5% Digitonin (BN2006, ThermoFisher)

Pierce Protease inhibitor mini tablets (A32953, ThermoFisher)

Nuclease-free water (AM9937, ThermoFisher)

DAPI (D1306, ThermoFisher)

Tagment DNA enzyme and small buffer kit (20034197, Illumina)

IDT for Illumina Nextera DNA Unique Dual Indexes Set A (96 Indexes, 96 Samples) (20027213, Illumina)

Phusion High fidelity PCR master mix with HF buffer (M0531L, New England Biolabs)

SsoAdvanced Universal SYBR green Supermix (1725271, BioRad)

MinElute PCR purification kit (28004, Qiagen)

AMPure XP magnetic beads (A63880, Beckman Coulter)

200 proof ethanol, anhydrous (459836, Sigma Aldrich)

BUFFERS

| **Wash buffer** | **Stock concentration** | **Final concentration** | **Volume: 10 mL** |
| --- | --- | --- | --- |
| Tris-HCl, pH 7.4 | 1 M | 10 mM | 100 μL |
| NaCl | 5 M | 10 mM | 20 μL |
| MgCl2 | 1 M | 3 mM | 30 μL |
| BSA | 10% | 1% | 1 mL |
| Tween-20 | 100% | 0.1% | 10 μL |
| Nuclease-free water |  |  | to 10 mL |

| **Lysis buffer** | **Stock concentration** | **Final concentration** | **Volume: 10 mL** | **Volume: 50 mL** |
| --- | --- | --- | --- | --- |
| Tris-HCl, pH 7.4 | 1 M | 10 mM | 100 μL | 500 μL |
| NaCl | 5 M | 10 mM | 20 μL | 100 μL |
| MgCl_2_ | 1 M | 3 mM | 30 μL | 150 μL |
| BSA | 10% | 1% | 1 mL | 5 mL |
| Tween-20 | 100% | 0.1% | 10 μL | 50 μL |
| NP-40 substitute | 100% | 0.1% | 10 μL | 50 μL |
| Digitonin | 5% | 0.1% | 100 μL | 500 μL |
| Nuclease-free water |  |  | to 10 mL | to 50 mL |

EQUIPMENT

Dounce homogenizers, 2 mL (KT885300-0002, VWR) or 7 mL (KT885300-0007, VWR)

Fly sieves (57333-965, VWR)

Corning 40 μm Cell strainers (07-201-430, Fisher Scientific)

Wide-bore pipette tips (1011-9410, USA Scientific)

Sorvall ST16R swinging bucket centrifuge (ThermoFisher) or equivalent

FACS Aria 4-color flow cytomter (BD Biosciences) or equivalent

T100 thermocycler (BioRad) or equivalent

ABI 7900HT qPCR system (ThermoFisher) or equivalent

Agencourt SPRIPlate 96R Super Magnet Plate (A32782, Beckman Coulter)

HiSeq 2500 or NovaSeq 6000 instrument (Illumina)

PROTOCOL

2-3 weeks prior to nucei isolation

*Fly crosses*

Set up fly crosses ~2 weeks before pilot or main experiment using target of interest *(TOI)-Gal4* x *UAS-Stinger-nls* flies (BL84277, Bloomington Stock Center). Flies should be reared on standard cornmeal agar at 25 °C.

**Note:** One bottle yields ~250-400 flies.

**Note:** 25 °C is standard rearing temperature. Rearing at a cooler temperature (18 or 22 °C) is useful to slow development or to grow difficult fly lines. Rearing at a higher temperature (28 °C) can drive higher GFP expression.

Day 1

*Preparation for nuclei isolation*

1. Sterilize Dounce homogenizer and A and B pestles (one set per sample).
2. Assemble fly sieves. Use three chambers separated by an upper coarse (25 mesh size, ~ 1 mm holes) and lower fine (24 mesh size, ~ 300 μm holes) mesh.
3. Place fly sieves at -80 °C at least 1 hour prior to the experiment.
4. Prepare 10 mL 10% BSA in 1X PBS.
5. Prepare 50 mL 1% BSA in 1X PBS
6. Prepare buffers 1 day prior to the experiment and store at 4 °C. Each sample requires 10 mL wash buffer and 7 mL lysis buffer.

*Nuclei isolation*

1. Add 5% digitonin and protease inhibitor tablets (1 tablet per 50 mL) to lysis buffer. Mix on nutator to prevent bubbles from forming.
2. Make sure centrifuge is pre-chilled to 4 °C.
3. Gather all reagents and place on ice in a large container.
4. Pre-chill homogenizers and pestles on ice.

**Note:** Perform all homogenization and isolation steps on ice.

1. Rinse homogenizers with 1% BSA in 1X PBS.
2. Place pestles in a sterilized container and soak in 1% BSA in 1X PBS.
3. Add lysis buffer (2 or 7 mL, depending on homogenizer size) with digitonin and protease inhibitor cocktail to each homogenizer.
4. Collect flies into a dry bottle (without food).

**Note:** Include a sample using flies that do not express GFP and process the sample in the same manner as the other samples. This sample will be used to set the gating scheme for flow cytometry.

1. Freeze flies for 5 min at -80 °C, one bottle at a time.
2. Remove flies from freezer and vortex at max speed for 10-15 seconds.
3. Transfer flies into fly sieve.
4. Shake sieve for 1 min to separate the fly heads from everything else. The bodies will remain in the upper chamber, while the heads will be in the middle chamber.
5. Gently tap one side of the sieve containing the heads on the table to cause the heads to fall to the side of the sieve. Transfer the fly heads into a homogenizer (containing lysis buffer) using a paint brush.
6. Repeat steps 9-13 for all samples while keeping the samples on ice.
7. Homogenize heads with the A (loose) pestle until resistance disappears (~20 strokes).
8. Pass homogenate through a 40 µm cell filter in a 1% BSA-cured 50 mL conical tube.
9. Rinse homogenizers thoroughly with nuclease-free water.
10. Transfer filtered homogenate to 1% BSA-cured Dounce homogenizer and homogenize with 15 slow strokes of the B (tight) pestle.

**Note:** Homogenizing the sample too fast or for too long will disrupt the intact nuclei.

1. Cure 14-mL round-bottom tubes with 1% BSA in 1X PBS. Discard BSA.
2. Transfer homogenate to cured 14-mL collection tube.
3. Repeat steps 15-20 for all samples while keeping the samples on ice.
4. Add 5 mL wash buffer to homogenates.
5. Centrifuge samples for 10 min, 500 x *g*, 4 °C.
6. Carefully remove supernatant without disturbing the pellet. Discard supernatant.
7. Add 3 mL wash buffer.
8. Centrifuge samples for 10 min, 500 x *g*, 4 °C.
9. Carefully remove supernatant and discard.
10. Resuspend pellet in 1 mL wash buffer by pipetting using a wide-bore pipette tip.
11. Add 3 μM DAPI to each tube.
12. Collect nuclei by flow cytometry. Be sure to set the sorting gates to collect DAPI+/GFP+ nuclei.

**Note:** Some flow cytometers require sample tubes to be a specific size (i.e. 5-mL round bottom tubes). If possible, perform all wash steps in an appropriate sample tube to minimize sample loss.

*Tagmentation*

1. Centrifuge isolated nuclei for 10 min, 500 x *g*, 4 °C.
2. Remove supernatant and resuspend nuclei in reaction buffer.

*Tagmentation reaction mix (per sample)*

25 μL 2X TD buffer (20 mM 1M Tris-HCl pH 7.6, 10 mM 1M MgCl_2_, 20% dimethyl formamide, and sterile water)

2.5 μL Tn5 enzyme

16.5 μL sterile PBS

0.5 μL 1% digitonin

0.5 μL 10% Tween-20

5 μL sterile water

1. Incubate samples for 23 min at 37 °C while shaking.
2. Purify tagmented DNA with a Qiagen MinElute PCR purification kit according to the kit instructions.

**Note:** This is a safe stopping point. Store samples at -20 °C overnight.

Day 2

*Amplification*

1. Mix tagmented DNA with reaction mix.

*PCR reaction mix (main reaction)*

20 μL tagmented DNA

2.5 μL Nextera index primer 1

2.5 μL Nextera index primer 2

25 μL Phusion High Fidelity Master Mix

1. Amplify DNA for 5 cycles:
2. Remove 5 μL amplified DNA and mix with qPCR reaction mix. Store the remaining 45 μL at 4 °C.

*qPCR side reaction*

5 μL amplified DNA from main reaction

0.5 μL Nextera index primer 1

0.5 μL Nextera index primer 2

5 μL SsoFast Eva Green Master Mix

4 μL sterile water

1. Perform qPCR mixture for 40 cycles.
2. Calculate the number of cycles corresponding to 25% maximum fluorescence from the qPCR reaction.

**Note:** In our experience, most samples containing 60,000 nuclei require 8-9 additional PCR cycles. Samples with fewer nuclei may require additional cycles.

1. Amplify the remaining 45 μL aliquot for the calculated number of PCR cycles.

*Clean-up*

1. Prepare fresh 80% ethanol.

**Note:** Ethanol prepared earlier will absorb water, which will decrease the actual ethanol concentration. 80% ethanol is critical for efficient purification.

1. Mix tagmented, amplified DNA with 0.5X (22.5 μL) AMPure XP beads by pipetting up and down 10 times. Incubate at room temperature for 10 minutes.
2. Place samples on a magnet plate for 5 min.
3. Remove supernatant and transfer to a new tube.
4. Add 1.1X (49.5 μL) AMPure XP beads. Mix by pipetting up and down 10 times. Incubate at room temperature for 10 min.
5. Place samples on magnet plate for 5 min.
6. Remove supernatant and discard.
7. Wash beads with 80% ethanol by pipetting the ethanol over the beads 10 times.
8. Place samples in magnet plate for 5 min.
9. Remove ethanol and let sample dry for 10 minutes
10. Mix beads with 20 μL 10 mM Tris-HCl, pH 8.5 by pipetting up and down 10 times. Incubate at room temperature for 10 min.

**Note:** We typically use the EB buffer included in the MinElute PCR purification kit.

1. Place samples on magnet plate for 5 min.
2. Carefully transfer supernatant to a new tube. Store samples at -20 °C until ready to sequence.
